# Biological Evaluation of Oxindole Derivative as a Novel Anticancer Agent against Human Kidney Carcinoma Cells

**DOI:** 10.3390/biom10091260

**Published:** 2020-08-31

**Authors:** Prasanta Dey, Amit Kundu, Sang Hoon Han, Kyeong-Seok Kim, Jae Hyeon Park, Sungpil Yoon, In Su Kim, Hyung Sik Kim

**Affiliations:** School of Pharmacy, Sungkyunkwan University, Suwon 16419, Korea; deyprasantadey@yahoo.com (P.D.); amitjupcl@gmail.com (A.K.); sang0306@nate.com (S.H.H.); caion123@nate.com (K.-S.K.); sky3640@naver.com (J.H.P.); syoon88@gmail.com (S.Y.)

**Keywords:** oxindole derivative, apoptosis, autophagy, glycolysis, tumor xenograft

## Abstract

Renal cell carcinoma has emerged as one of the leading causes of cancer-related deaths in the USA. Here, we examined the anticancer profile of oxindole derivatives (SH-859) in human renal cancer cells. Targeting 786-O cells by SH-859 inhibited cell growth and affected the protein kinase B/mechanistic target of rapamycin 1 pathway, which in turn downregulated the expression of glycolytic enzymes, including lactate dehydrogenase A and glucose transporter-1, as well as other signaling proteins. Treatment with SH-859 altered glycolysis, mitochondrial function, and levels of adenosine triphosphate and cellular metabolites. Flow cytometry revealed the induction of apoptosis and G0/G1 cell cycle arrest in renal cancer cells following SH-859 treatment. Induction of autophagy was also confirmed after SH-859 treatment by acridine orange and monodansylcadaverine staining, immunocytochemistry, and Western blot analyses. Finally, SH-859 also inhibited the tumor development in a xenograft model. Thus, SH-859 can serve as a potential molecule for the treatment of human renal carcinoma.

## 1. Introduction

Renal cell carcinoma (RCC) is categorized as the 6th and 10th most common cancer in males and females in the USA, respectively [1]. Approximately 65,340 new cases were diagnosed and 14,970 people died from RCC in the USA in 2018 [2], with it being considered the most hostile type [3,4]. Poor outcomes were observed in patients with clear cell renal cell carcinoma (ccRCC) showing distant metastasis, with a near 12% survival rate in 5 years [5], and approximately 20–40% of patients suffer from distant metastasis [6].

The discovery and development of new therapeutic agents are necessary to overcome the resistance of cancer cells to chemotherapy and radiation therapy [7]. Therefore, it is critical to identify a novel molecule that can check cancer metastasis and offer direction for emerging novel therapeutic strategies and clinical decision making. The development of targeted therapeutics against the mechanistic target of rapamycin 1 (mTOR) and tyrosine kinase (TK) has been a foremost breakthrough in anticancer drug discovery against ccRCC [7]. New therapeutic approaches have emerged as effective treatment possibilities against advanced ccRCC by inducing apoptosis and autophagy and targeting cancer cell metabolism [8].

Apoptosis, or programmed cell death, is a highly regulated and conserved cellular phenomenon, maintained by a highly organized network of cell-intrinsic suicide machinery [9]. When homeostasis between cell proliferation and death changes, alterations to apoptosis-inducing pathways occur that result in oncogenesis [10]. Receptor-mediated extrinsic pathways or mitochondria-mediated intrinsic pathways can trigger apoptosis. Additionally, other cellular organelles, including the endoplasmic reticulum, cytoskeleton, lysosomes, and nucleus, may participate in apoptotic signaling by detecting impairment or integrating pro-apoptotic signals [11]. On the contrary, autophagy is a highly controlled cell-survival pathway regulated by autophagy-related proteins conserved in all eukaryotes. This process includes the degradation of cellular pieces of machinery, including proteins and damaged cytoplasmic organelles, which ultimately results in the recovery of nutrients and the production of energy [12]. Therefore, targeting both these cellular pathways can lead to a potential therapeutic strategy.

Cancer cells need enormous amounts of energy for their growth and survival. Although a plentiful amount of oxygen is available, cancer cells catabolize glucose sequentially through a series of metabolic reactions comprising glycolysis and cellular respiration through oxidative phosphorylation [13] and convert glucose to lactate to meet their high energy demand [14,15]. Human kidney cancer cells upregulate the expression of glycolytic enzymes and increase their glucose uptake, lactate synthesis, and autophagy suppression, thus hastening oncogenic growth.

The oxindole backbone is a heterocycle found in various biologically active synthetic compounds and natural products [16,17]. Notably, spiro-oxindole and 3-substituted compounds have a vast range of biological potential such as anti-inflammatory, anti-bacterial, and anti-tumor activities [18,19,20]. Hence, synthesis of novel, biologically active oxindole derivatives has received a large amount of interest [21,22]. In this study, we evaluated the anticancer effect of a small molecule (SH-859). We studied how SH-859 inhibits the progression of ccRCC tumorigenesis and the mechanism underlying its anticancer activity against the human kidney cancer cell line, 786-O. Our data show that SH-859 treatment significantly inhibited the viability of human kidney cancer cells (786-O) as compared to that of normal kidney cells (NRK52E). Furthermore, SH-859 treatment induced apoptosis and autophagy and inhibited the production of major cellular metabolites. Our results clearly show that SH-859 can serve as a potential therapeutic agent for RCC.

## 2. Materials and Methods

### 2.1. Chemicals and Reagents

Roswell Park Memorial Institute 1640 (RPMI-1640) (cat. no. LM011-01) and penicillin/streptomycin (cat. no. LS202-02) were obtained from Welgene Co. (Gyeongsan-si, Gyeongsangbuk-do, South Korea). Trypsin (cat. no. 25300-054), Dulbecco’s phosphate-buffered saline (cat. no. 31600-026), and fetal bovine serum (FBS; cat. no. 10099-141) were purchased from Gibco, Life Technologies (Carlsbad, CA, USA). Pierce bicinchoninic acid protein assay reagent A and B (cat. no. 23228 and 1859078) were purchased from Thermo Fisher Scientific (Waltham, MA, USA). Goat anti-mouse IgG (H + L) cross-adsorbed secondary antibody, rhodamine Red-X (cat. no. R6393), was acquired from Invitrogen (Carlsbad, CA, USA). Acridine orange solution (cat. no. A8097), monodansylcadaverine (MDC) (cat. no. D4008), acetone (cat. no. 270725), goat serum (cat. no. G9023), Triton X-100 (cat. no. T8787), thiamine (cat. no. T1270), 4,6-diamidino-2-phenylindole dihydrochloride (DAPI; cat. no. D9542), pyruvic acid (cat. no. 107360), and 3-(4,5-dimethyl-2-thiazolyl)-2,5-diphenyl-2H-tetrazolium bromide (MTT; cat. no. M5655) were supplied by Sigma-Aldrich (St. Louis, MO, USA). The protein extraction solution, PRO-PREP (cat. no. 17081), was purchased from Intron Biotechnology. Maleic acid (cat. no. PHR1273), α-ketoglutaric acid (cat. no. 75890), succinic acid (cat. no. 14078), and fumaric acid (cat. no. 47910) were purchased from Fluka (St. Louis, MO, USA). Bovine serum albumin (BSA) (cat. no. BSAS 0.1) and crystal violet (cat. no. C1035) were purchased from Bovogen, Biosesang Inc (Seongnam-si, Gyeonggi-do, South Korea). Immobilon Forte Western HRP substrate (cat. no. WBLUF0100) and polyvinylidene difluoride (PVDF) membrane (cat. no. IPVH00010) were procured from Millipore (Gangnam-GU, Seoul, South Korea). Lactic acid (cat. no. L0226) was procured from Tokyo Chemical Industry Co Ltd (Tokyo, Japan). The primary antibodies for PKM2 (cat. no. 4053), mTOR (cat. no. 2983), Bax (cat. no. 5023), LDHA (cat. no. C4B5), cytochrome c (cat. no. 4280), GLUT1 (cat. no. 12939), beclin 1 (cat. no. 3495), ATG7 (cat. no. 8558), LC3A/B (cat. no. 12741), LC3B (cat. no. 83506), cleaved caspase 3 (cat. no. 9664), glyceraldehyde 3-phosphate dehydrogenase (GAPDH; cat. no. 5174), cleaved caspase 9 (cat. no. 20750), cleaved PARP (cat. no. 5625), AKT (cat. no. 9272), phosphorylated AKT (p-AKT; S473) (cat. no. 9271), and phosphorylated mTOR (p-mTOR; S2448) (cat. no. 2971) were obtained from Cell Signaling Technology, Inc. (Danvers, MA, USA), while those against c-Myc (cat. no. sc-789), Bcl2 (cat. no. SC-7382), cyclin A (cat. no. SC-271682), cyclin B (cat. no. SC-166210), cyclin D (cat. no. SC-20044), HIF1α (cat. no. SC-13515), and MCT-4 (cat. no. SC-376140) were supplied by Santa Cruz Biotechnology, Inc (Dallas, TX, USA). Seahorse XF Cell Mito stress test kit (cat. no. 103015-100), Seahorse XF glycolytic rate assay kit (cat. no. 103344-100), and Seahorse XF real-time ATP rate assay kit (cat. no. 103592-100) were purchased from Agilent Technologies (Santa Clara, CA, USA). Commercially available reagents were used without additional purification unless otherwise stated. Sealed tubes (13 × 100 mm^2^) were purchased from Fischer Scientific, dried in the oven overnight, and cooled to room temperature before use. Thin-layer chromatography was carried out using plates coated with Kieselgel 60 F254 (Merck). For flash column chromatography, E. Merck Kieselgel 60 (230–400 mesh) was used. Nuclear magnetic resonance spectra (^1^H and ^13^C NMR) were recorded on a Bruker Unity 400 spectrometer (Bruker, MA, USA) and 500 spectrometer using CDCl_3_ and DMSO-d_6_ solution; chemical shifts were reported as parts per million (ppm). Resonance patterns were reported with the notations s (singlet), d (doublet), t (triplet), q (quartet), and m (multiplet). Moreover, the notation bar was used to indicate a broad signal. Coupling constants (J) are reported in hertz (Hz). IR spectra were recorded on a Varian 2000 Infrared spectrophotometer and were reported as cm^−1^. High-resolution mass spectra were recorded on a JEOL JMS-600 spectrometer.

### 2.2. Preparation of the Oxindole Derivative (SH-859)

**Scheme 1:**
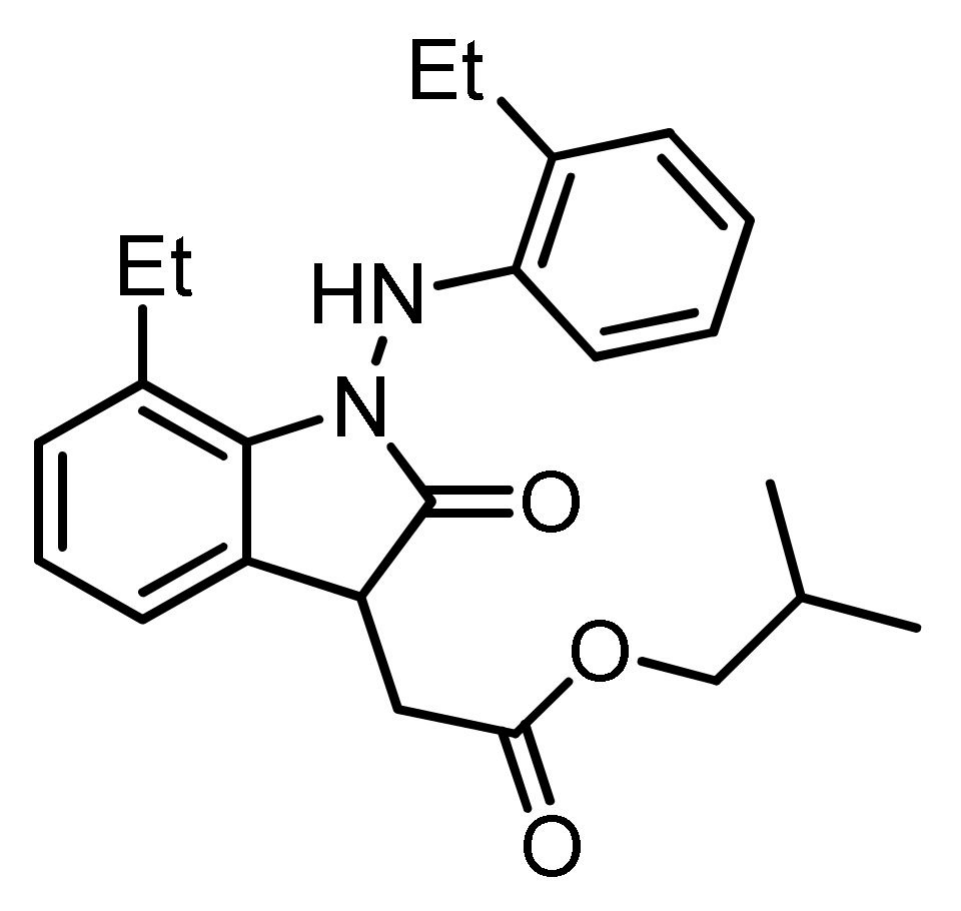
Isobutyl 2-(7-ethyl-1-((2-ethylphenyl)amino)-2-oxoindolin-3-yl)acetate (SH-859) [23].

**Scheme 2:**
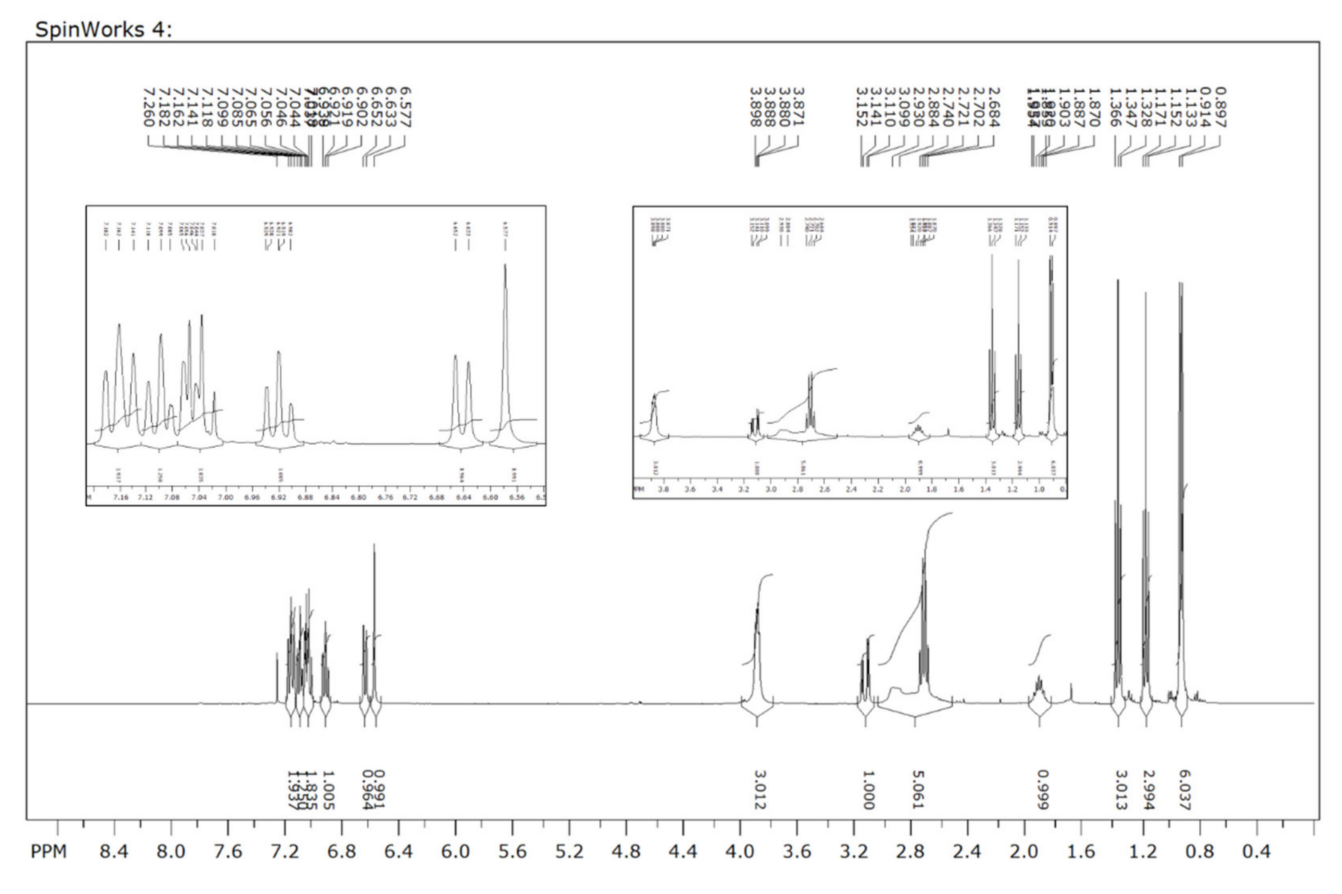
^1^H NMR spectroscopic data of SH-859 [23].

**Scheme 3:**
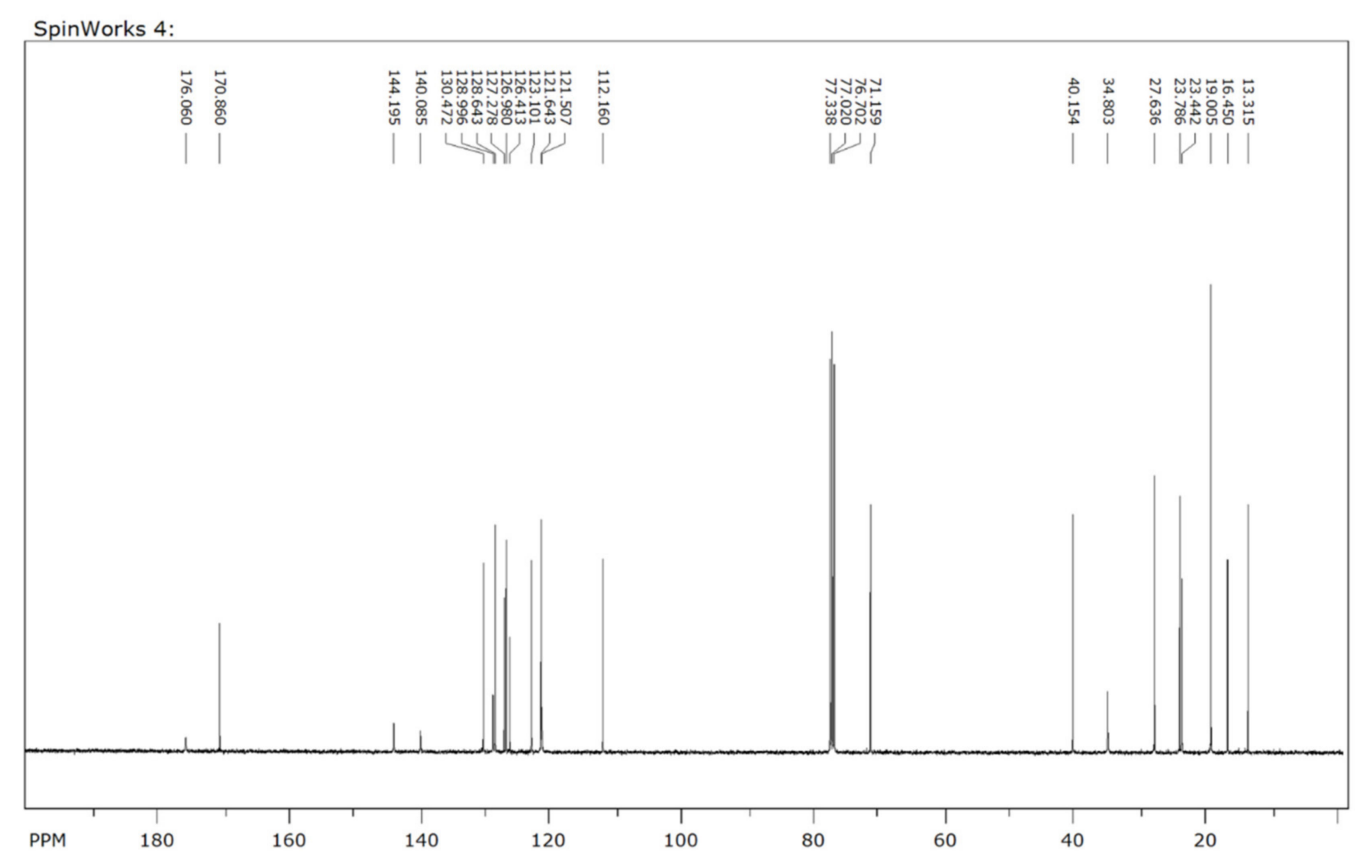
^13^C NMR spectroscopic data of SH-859 [23].

To an oven-dried sealed tube, charged with 1,2-bis(2-ethylphenyl)diazene (47.7 mg, 0.2 mmol, 100 mol %), [RhCp*Cl2]2 (3.1 mg, 0.005 mmol, 2.5 mol %), AgSbF6 (6.9 mg, 0.02 mmol, 10 mol %), and Acetic acid (AcOH) (12.0 mg, 0.2 mmol, 100 mol %), we added diisobutyl fumarate (68.5 mg, 0.3 mmol, 150 mol %) and Dichloroethane (DCE) (0.5 mL) under air at room temperature. The reaction mixture was stirred for 16 h at 80 °C. Thereafter, the reaction mixture was cooled to room temperature, and Zn powder (65.4 mg, 1.0 mmol, 500 mol %), AcOH (60.0 mg, 1.0 mmol, 500 mol %), and ethanol (EtOH) (1 mL) were added to the resulting reaction mixture without further purification or work-up. The reaction mixture was stirred for 12 h at room temperature, filtered, washed with dichloromethane (20 mL), and concentrated in vacuo. The residue was purified by flash column chromatography (*n*-hexane/EtOAc = 1:1) to obtain a yield of 73% SH-859 (57.7 mg) (Scheme 1, Scheme 2 and Scheme 3) [23].

### 2.3. Characterization Data for SH-859

57.7 mg (73%); brown sticky solid; ^1^H NMR (400 MHz, CDCl3) δ 7.18–7.01 (m, 5H), 6.91 (t, J = 7.2 Hz, 1H), 6.64 (d, J = 7.6 Hz, 1H), 6.57 (s, 1H), 3.89–3.87 (m, 2H), 3.11 (dd, J = 16.8, 4.4 Hz, 1H), 2.93–2.68 (m, 5H), 1.93–1.87 (m, 1H), 1.34 (t, J = 7.6 Hz, 3H), 1.15 (t, J = 7.6 Hz, 3H), 0.89 (d, J = 6.8 Hz, 3H); 13C NMR (100 MHz, CDCl3) δ 176.0, 170.8, 144.2, 140.1, 130.4, 128.9, 128.6, 127.2, 126.9, 126.4, 123.1, 121.6, 121.5, 112.1, 71.1, 40.1, 34.8, 27.6, 23.8, 23.4, 19.0, 16.4, 13.3; IR (KBr) υ 3291, 3061, 2963, 2931, 2873, 2362, 1728, 1604, 1504, 1455, 1378, 1265, 1188, 1159, 1063, 997, 943, 737 cm^−1^; high-resolution mass spectra (Orbitrap, ESI) calcd for C_24_H_31_N_2_O_3_ [M + H]^+^ 395.2329 was found to be 395.2256.

### 2.4. Cell Lines and Cell Culture

The human kidney carcinoma cell line, 786-O, and human normal kidney cell line, NRK52E, were purchased from the American Type Culture Collection (Manassas, VA, USA). The cells were maintained in RPMI-1640 medium supplemented with 10% heat-inactivated FBS, 100 U/mL penicillin, and 100 μg/mL streptomycin at 37 °C in a humidified atmosphere of 5% CO_2_. Cells collected at the logarithmic growth phase were used for all experiments.

### 2.5. Cytotoxicity Assay

The MTT reagent was used to determine the viability and IC_50_ of 786-O and NRK52E cells, as previously described [24]. The 786-O and NRK52E cells were seeded and treated with the small molecules at concentrations ranging from 5 to 50 μM for 48 h. Thereafter, the MTT solution and DMSO were added to the cells. A VERSA Max Microplate Reader (Molecular Devices Corp., Silicon Valley, CA, USA) was used to measure the optical density at 540 nm. The data obtained from three independent experiments were used to calculate IC_50_ from sigmoidal dose-response curves, using SigmaPlot 10 software (Jandel Scientific, San Rafael, CA, USA).

### 2.6. Analysis of Cell Cycle Progression

After 786-O cells reached 80% confluency, they were treated with the desired concentration of SH-859 for 48 h. All the cells were collected and stained with propidium iodide (PI, 10 μg/mL) using the Cycletest Plus DNA Reagent Kit (BD Biosciences, San Jose, CA, USA). Cell cycle progression was evaluated by flow cytometry (Guava EasyCyte flow cytometer; Millipore, Billerica, MA, USA).

### 2.7. Annexin V/PI Double Staining

The 786-O cells were treated with the desired concentration of SH-859 for 48 h, transferred to flow cytometry tubes, and treated with annexin V-Fluorescein isothiocyanate (FITC) and PI for 5 min in the dark. Apoptotic cells were detected by flow cytometry.

### 2.8. Western Blot Analysis

Western blot analysis was performed as previously described [25]. The 786-O cells were treated with the indicated concentration of the small molecule for 48 h. Cells were harvested and the whole cell lysate was prepared using the PRO-PREP cell lysis buffer. The lysate was electrophoresed on a 6–12% gel using sodium dodecyl sulfate (SDS) polyacrylamide gel electrophoresis (PAGE). Thereafter, the separated protein bands were transferred onto PVDF membranes. Subsequently, the membranes were blocked with a blocking buffer and incubated with primary and corresponding secondary antibodies. The blots were developed using the Immobilon Forte Western HRP substrate.

### 2.9. Acridine Orange Staining

Acridine orange staining was carried out as previously described [26]. The 786-O cells were seeded and treated with the small molecules at the appropriate concentration for 48 h after reaching the required confluency. Thereafter, the cells were treated with 1 μg/mL of acridine orange (2.7 μM) in a serum-free medium at 37 °C for 15 min, at room temperature. Subsequently, the cells were washed with phosphate buffer saline (PBS) and acidic vesicular organelles were examined under a fluorescence microscope (FV10i; Olympus Corp., Tokyo, Japan) at 400× magnification. Bright green fluorescence was observed in the cytoplasm and nucleus of the stained cells; acidic vesicular organelles emitted bright red color.

### 2.10. MDC Staining

MDC staining was performed as described previously [26]. After treatment for 48 h, the formation of autophagolysosomes was detected by incubating the cells with the lysosomotropic autofluorescent compound, MDC (50 μM), at 37 °C for 15 min. Thereafter, the cells were washed with PBS and immediately examined under a fluorescence microscope (FV10i; Olympus Corp., Tokyo, Japan) at 400× magnification.

### 2.11. Immunofluorescence Analysis

After treatment with the small molecule, the 786-O cells were fixed with acetone, incubated with primary antibodies against LC3B (1:400), and subsequently treated with Alexa Fluor-conjugated secondary antibodies (1:200) and DAPI (0.1 μg/mL), using standard protocols. Cells were examined under a fluorescence microscope (FV10i; Olympus Corp., Tokyo, Japan) at 400× magnification.

### 2.12. Pyruvate Kinase Activity Assay

The pyruvate kinase activity in 786-O cells after SH-859 treatment was determined using a kit from Abcam (ab83432), according to the manufacturer’s instructions. Control and treated cells were lysed with the assay buffer and transferred to a 96-well plate. Thereafter, 50 μL of the master mix was added to each sample. The optical density (OD 570 nm) was determined as a function of time using a plate reader. All samples were measured in triplicate.

### 2.13. Analysis of Glycolysis, Glycolytic Intermediates, and Mitochondrial Activity

Cellular bioenergetic markers, such as oxygen consumption rate (OCR), proton efflux rate (PER), and ATP release rate, were examined with the Seahorse XF96 Flux Analyzer (Seahorse Bioscience, Billerica, MA, USA), according to the manufacturer’s instructions [27]. Briefly, medium from SH-859-treated cells was replaced with Agilent Seahorse XF assay medium, and the cells were incubated for 45–60 min in a CO_2_-free, 37 °C incubator. The cells were subjected to the Seahorse XF Cell Mito Stress Test, XF Glycolytic Rate assay, and XF Real-Time ATP rate assay. All measurements were done at certain time intervals, as per the manufacturer’s instructions.

High-performance liquid chromatography (HPLC) (Gilson, France) has been used to quantify the levels of pyruvate and lactate [8]. Here, a liquid chromatography (LC) system, containing an LC-321/322/350 pump (Gilson, France), an autosampler (Gilson-234), and a UV/VIS-151 detector (Gilson), was used. The Synergi Hydro-RP C18 column (250 × 4.6 mm, 4 µm, 80 Å; Phenomenex, Torrance, CA, USA), preceded by a pre-column (Phenomenex), was used for the detection and quantification of samples. Flow rates of 0.7 and 0.8 mL/min were maintained for pyruvate and lactate, respectively. Here, an isocratic mobile phase of water with 20 mM potassium phosphate and water with 0.1% phosphoric acid were used for pyruvate and lactate, respectively. All the samples were carefully mixed with acetonitrile, containing thiamine (internal standard), and centrifuged. The supernatants were analyzed with HPLC (LC-321/322/350 pump) at 220 and 210 nm for pyruvate and lactate, respectively.

### 2.14. Colony Formation Assay

The 786-O cells were seeded in a 6-well plate (1000 cells per well) for 48–72 h, followed by treatment with different concentrations (5, 10, and 20 µM) of SH-859. The growth medium and the small molecule were replaced every 3 days for 2 weeks. The cells were washed with PBS, fixed in formalin (10%), and stained with crystal violet (0.1%). Colonies were counted and normalized to the number in the control group, as previously described [28,29].

### 2.15. Tumor Xenograft Study

Male BALB/c nude mice (5 weeks old) were bought from Central Lab Animal Inc. (Hamamatsu, Japan). All animals were housed at 22 ± 11 °C and 50–60% relative humidity, with a 12-h light/dark cycle. The Sungkyunkwan University (SKKU) Animal Care Committee approved this experimental procedure (SKKU-2018-0065). For the formation of tumors, 786-O cells (5 × 10^6^) in FBS-free RPMI medium were mixed with an equal volume of Matrigel and inoculated subcutaneously into the right front axilla of the mouse. Tumor sizes were measured every third day, using calipers. Tumor volumes were calculated as per the following formula (0.5 × long axis × short axis^2^). After the tumor volume reached 150 cm^3^, the mice were divided randomly into three different groups. The control group was left untreated. The test (SH-859) group of mice received SH-859 (5 mg/kg, intraperitoneally (i.p.), three times/week). The doxorubicin group of mice received doxorubicin (2 mg/kg, i.p., once/week). Tumor weight measurements was performed in each group.

### 2.16. Statistical Analysis

Data are expressed as the mean ± standard deviation (SD). Statistically significant differences between the groups were determined using analysis of variance (ANOVA) followed by Tukey’s multiple comparison tests. For all the tests, a significance level of 5% (*p* < 0.05) was used.

## 3. Results

### 3.1. SH-859 Prevented 786-O Cell Progression

**Figure 1:**
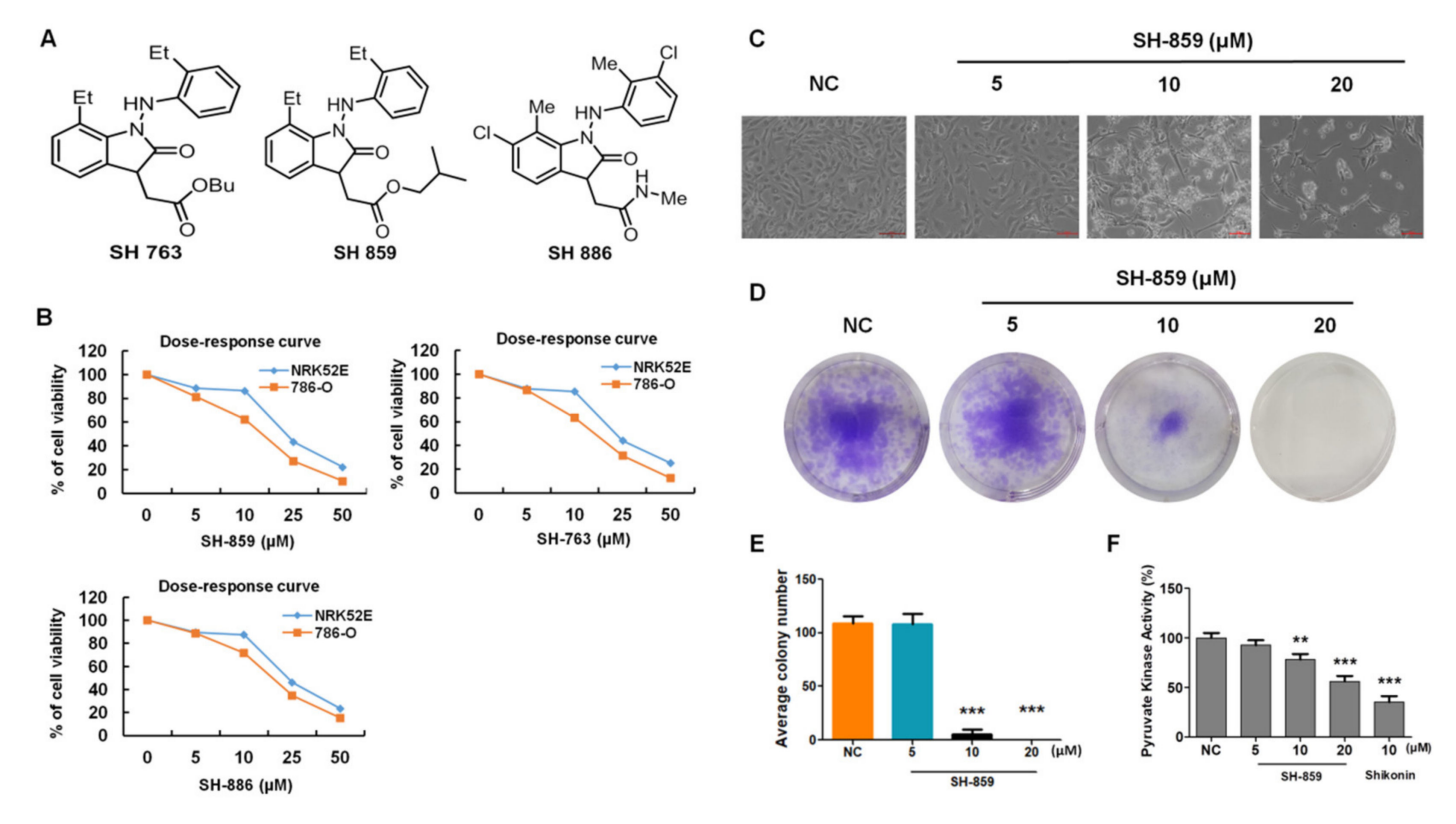
Consequences of oxindole derivative treatment on the survival, morphology, and colony formation ability of 786-O cells. (**A**) The chemical structures of various oxindole derivatives active against 786-O cells. (**B**) Dose–response curve of oxindole derivatives on the viability (determined using 3-(4,5-dimethyl-2-thiazolyl)-2,5-diphenyl-2H-tetrazolium bromide (MTT) assay) of kidney cancer 786-O cells and their subsequent treatment with indicated small molecules at concentrations ranging from 5–50 μM for 48 h. IC_50_ was determined by SigmaPlot software. Small molecules reduced the survival of 786-O cells as compared with normal control (untreated 786-O cells) and normal kidney cells (NRK52E). (**C**) The morphology of normal control (untreated 786-O cells) and SH-859-treated 786-O cells. The morphological variations were observed after oxindole derivative (SH-859) treatment as compared with normal control (untreated 786-O cells). (**D**) Images of the colony formation assay of 786-O cells treated with SH-859 in six-well plates. 786-O cell colonies were visually verified and counted under a light microscope. No differences in colony numbers were observed between the normal control (untreated 786-O cells) and the SH-859 (5 μM)-treated cells as compared with those treated with higher concentrations of SH-859. (**E**) Quantitative estimation of colony number. (**F**) Evaluation of SH-859 treatment on pyruvate kinase activity in 786-O cells. Shikonin (10 µM) was used as a positive control. Representative records of three independent experiments (*n* = 3) are shown. One-way ANOVA was used to compare the means of different concentrations. Differences between means were considered significant at *p* < 0.05 using Tukey’s multiple comparison test; ** *p* < 0.01 and *** *p* < 0.001 as compared with normal control (untreated 786-O cells). NC: normal control (untreated 786-O cells).

Han et al. (2017) have described the synthetic methodology of small molecules in detail [23]. To confirm the most effective small molecules against tumor progression, we treated 786-O cells with various small molecules. As shown in Figure 1A,B, and Figure S1, small molecule (SH-859, SH-763, and SH-886) treatment for 48 h significantly inhibited the viability of 786-O cells (SH-859, IC_50_-14.3 µM; SH-763, IC_50_-14.5 µM; and SH-886, IC_50_-16.7 µM) compared to NRK52E cells (SH-859, IC_50_-20.5 µM; SH-763, IC_50_-19.2 µM; and SH-886, IC_50_-20.9 µM). In the subsequent experiment, SH-859 was used as the experimental test molecule because of its lower IC_50_ value in 786-O cells and higher inhibitory concentration in NRK52E cells. Treatment with SH-859 not only lowered cell viability but also induced significant morphological changes in 786-O cells (Figure 1C). Additionally, we also examined the consequence of small molecule treatment on cell growth. In the colony formation assay, the number of colonies was higher in normal control (untreated 786-O cells) than in SH-859-treated cells. This small molecule inhibited the colony formation ability of 786-O cells in a concentration-dependent manner (Figure 1D,E). SH-859 was able to impair pyruvate kinase activity in 786-O cells at a concentration of cell proliferation inhibition, such as 10 and 20 μM (Figure 1F), which is comparable with shikonin (10 µM). This suggests that the inhibition of PKM2 by SH-859 was dependent on its effect on glycolysis.

### 3.2. Analysis of Cell Cycle Progression

**Figure 2:**
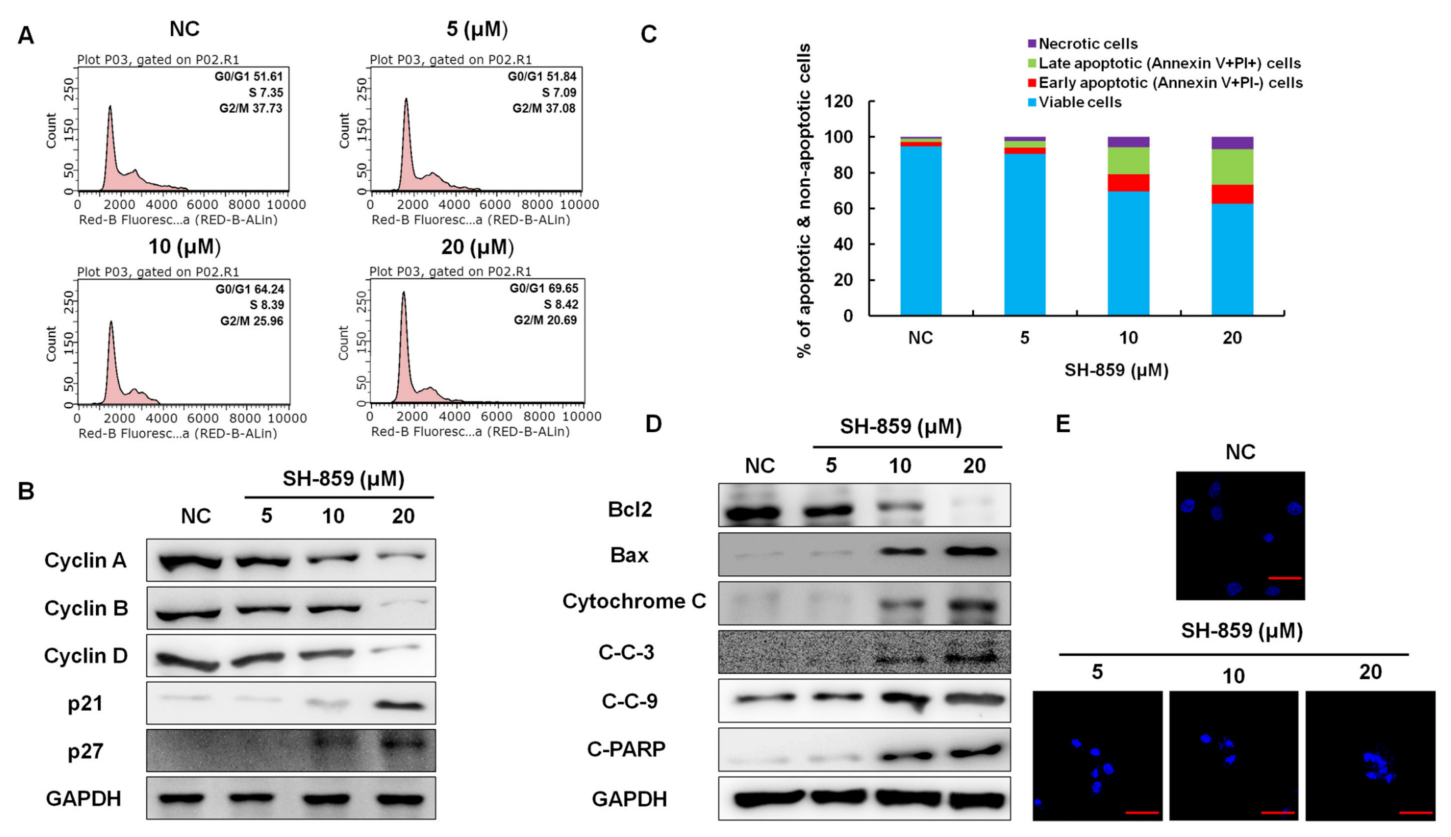
Effect of SH-859 on cell cycle and apoptosis regulation in 786-O cells. The cells were grown to log phase and treated with the specified concentrations of SH-859 (5, 10, and 20 μM) for 48 h. (**A**) To evaluate cell distribution at each phase of the cell cycle, we stained all the cells with propidium iodide and investigated them by flow cytometry. (**B**) Effect of SH-859 on the expression levels of different cell cycle regulatory proteins. The cells were harvested, and cell lysates were examined by Western blotting using antibodies specific for cyclin A, B, and D; P21; and P27. Glyceraldehyde 3-phosphate dehydrogenase (GAPDH) was used as an internal loading control. (**C**) The percentage of apoptotic cells was graphically represented after analysis by flow cytometry. (**D**) Effect of SH-859 on the expression levels of apoptosis regulatory proteins. The cells were harvested, and cell lysates were examined by Western blotting using antibodies specific for Bcl2, Bax, cytochrome c, cleaved caspase 3, cleaved caspase 9, and cleaved Poly (ADP-ribose) polymerase (PARP). GAPDH was used as an internal loading control. (**E**) Cells were stained with 4,6-diamidino-2-phenylindole dihydrochloride (DAPI) after SH-859 treatment. Images were observed using confocal microscopy (400×). Scale bars indicate 10 μm. Representative results of three independent experiments (*n* = 3) have been shown. NC: normal control (untreated 786-O cells).

To explore the effect of SH-859 on the cell cycle, we treated cells with SH-859 at a specific concentration for 48 h and assessed them using flow cytometry. No noteworthy change was observed after treatment with 5 μM of SH-859; however, a substantial rise in the G0/G1 phase cell population was detected after treatment with 10 or 20 μM of SH-859 (Figure 2A, Figure S2). Treatment with SH-859 significantly downregulated the expression of cyclin A/E, cyclin D, and cyclin B proteins (Figure 2B) as compared with the normal control (untreated 786-O cells). Consequently, the amount of p21 and p27 were also upregulated after SH-859 treatment in 786-O cells as compared with the normal control (untreated 786-O cells).

### 3.3. Induction of Cellular Apoptosis by SH-859

To analyze whether SH-859 induced apoptosis in 786-O cells, we performed flow cytometric analysis. The 786-O cells were treated with various concentrations of SH-859 and were doubled-stained with annexin V and PI. SH-859 increased annexin V^+^PI^−^ early- and annexin V^+^PI^+^ late-stage apoptotic cell death in a concentration-dependent manner (Figure 2C). Western blotting of proteins responsible for the regulation of apoptosis was performed to uncover the apoptosis-related mechanism. SH-859 was found to upregulate Bax expression and downregulate Bcl2 expression in a concentration-dependent manner, as compared with normal control (untreated 786-O cells) (Figure 2D). On the contrary, cytochrome c, cleaved caspase 3 (C-C-3), cleaved caspase 9 (C-C-9), and cleaved PARP (C-PARP) were upregulated after SH-859 treatment, which confirmed that SH-859 induces apoptotic cell death via a mitochondria-dependent pathway ((Figure 2D). DNA content is one of the most commonly used quantification techniques to assess the cell position in the cell cycle and also to evaluate the presence of apoptotic cells (characterized by fractional DNA content). Apart from the tagging of cells with annexin V and PI, apoptotic cells can also be recognized by the chromatin condensation that occurs during apoptosis. When the chromatin condensation occurs in the nucleus, the pixel intensity of DAPI increases. This chromatin condensation reduces the nucleus size, and, hence, the nuclear area [30,31,32,33]. Here, SH-859 treatment also induced nuclear condensation and DNA fragmentation, which was confirmed by DAPI staining (Figure 2E). Due to this widespread DNA fragmentation that occurs through apoptosis after SH-859 treatment, cells with the low-molecular DNA fragments can be recognized as the apoptotic cells with fractional DNA content.

### 3.4. SH-859 Induced Autophagy

**Figure 3:**
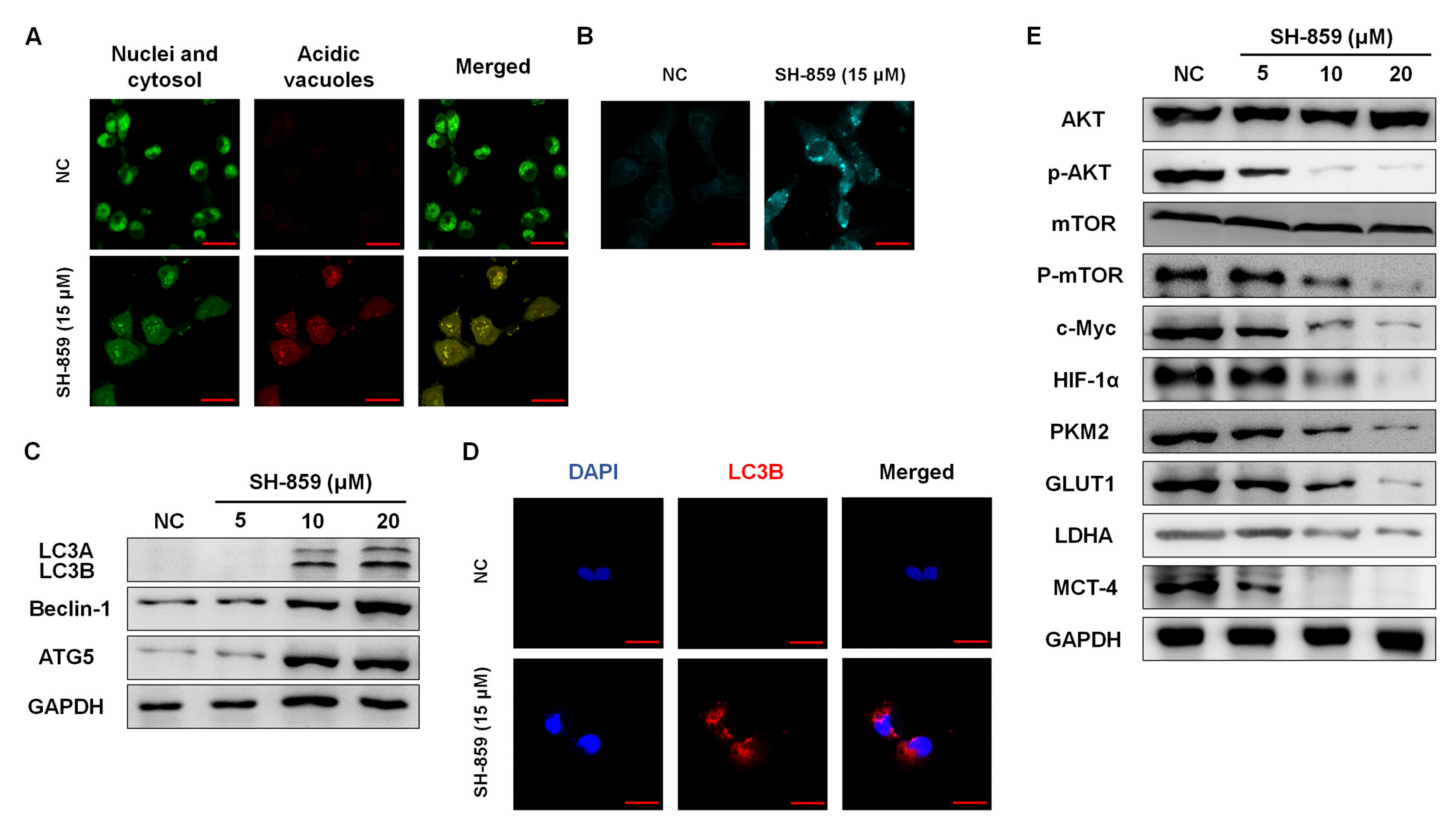
Evaluation of autophagy induction and various signaling pathways after SH-859 (15 μM) treatment for 48 h. (**A**) 786-O cells were treated with SH-859 (15 μM) and stained with acridine orange (1 μg/mL). The formation of acidic vesicular organelles was examined under the confocal microscope. Fluorescent green staining in the cytoplasm and nucleus and fluorescent red or orange-red staining of autophagic vacuoles was detected. (**B**) Examination of autolysosome development with MDC staining. 786-O cells were treated, stained with MDC (50 μM), and inspected using confocal microscopy. Scale bars indicate 50 μm. (**C**) Immunoblot analysis of autophagy regulatory proteins in 786-O cells. Western blotting of whole-cell lysates was performed using antibodies specific for light chain 3-I/II (LC3-I/II), ATG5, and Beclin 1. GAPDH was used as an internal loading control. Representative blots are shown. (**D**) Immunofluorescence analysis was performed to evaluate the expression of light chain 3B (LC3B) after SH-859 (15 μM) treatment. Higher LC3B expression (indicated by red fluorescence) was observed in SH-859-treated cells than in normal control (untreated 786-O cells). DAPI was used for nuclear staining. Goat anti-mouse IgG (H + L) cross-adsorbed secondary antibody Rhodamine Red-X was used to detect LC3B. (**E**) Expression levels of proteins associated with various signaling pathways after SH-859 treatment. Cells were treated with SH-859 and Western blotting was performed to examine the expression pattern of signaling pathway proteins. Representative blots are shown. Representative results from three independent experiments (*n* = 3) have been shown. NC: normal control (untreated 786-O cells).

PKM2 overexpression activates mammalian target of rapamycin complex 1 (mTORC1) (a key regulator of autophagy) and inhibits autophagy in various cancer cells [34,35,36]. To analyze whether SH-859 can activate autophagy in 786-O cells, we performed acridine orange and MDC staining. SH-859 significantly increased autophagosome and autophagolysosome formation (confirmed from the staining results with two acidotropic dyes, acridine orange and MDC) (Figure 3A,B). Autophagy-related proteins (LC3 I/II, Autophagy related 5 or ATG5, and Beclin 1) were also upregulated after SH-859 treatment (Figure 3C).

The high expression of light chain 3B (LC3B) (an autophagy marker) was detected in SH-859-treated cells in the ICC, which was consistent with immunoblot results (Figure 3D). To elucidate the signaling network implicated in autophagy regulation of SH-859-treated 786-O cells, we examined the Akt/mTOR pathway. mTOR is an important protein that maintains or regulates autophagy. Therefore, we evaluated the relationship between autophagy regulation and the role of the Akt/mTOR pathway in 786-O cells. We found that SH-859 considerably downregulated the expression of p-Akt, p-mTOR, and other associated proteins (c-Myc, HIF-1α, and PKM2) in a concentration-dependent manner, without affecting the corresponding total protein levels (Figure 3E). This finding indicated that the Akt/mTOR pathway may play an important role in the regulation of autophagy in 786-O cells and SH-859 may induce autophagy via the Akt/mTOR pathway.

### 3.5. SH-859 Attenuated Glycolysis, Cellular Metabolite Level, and Mitochondrial Activity

**Figure 4:**
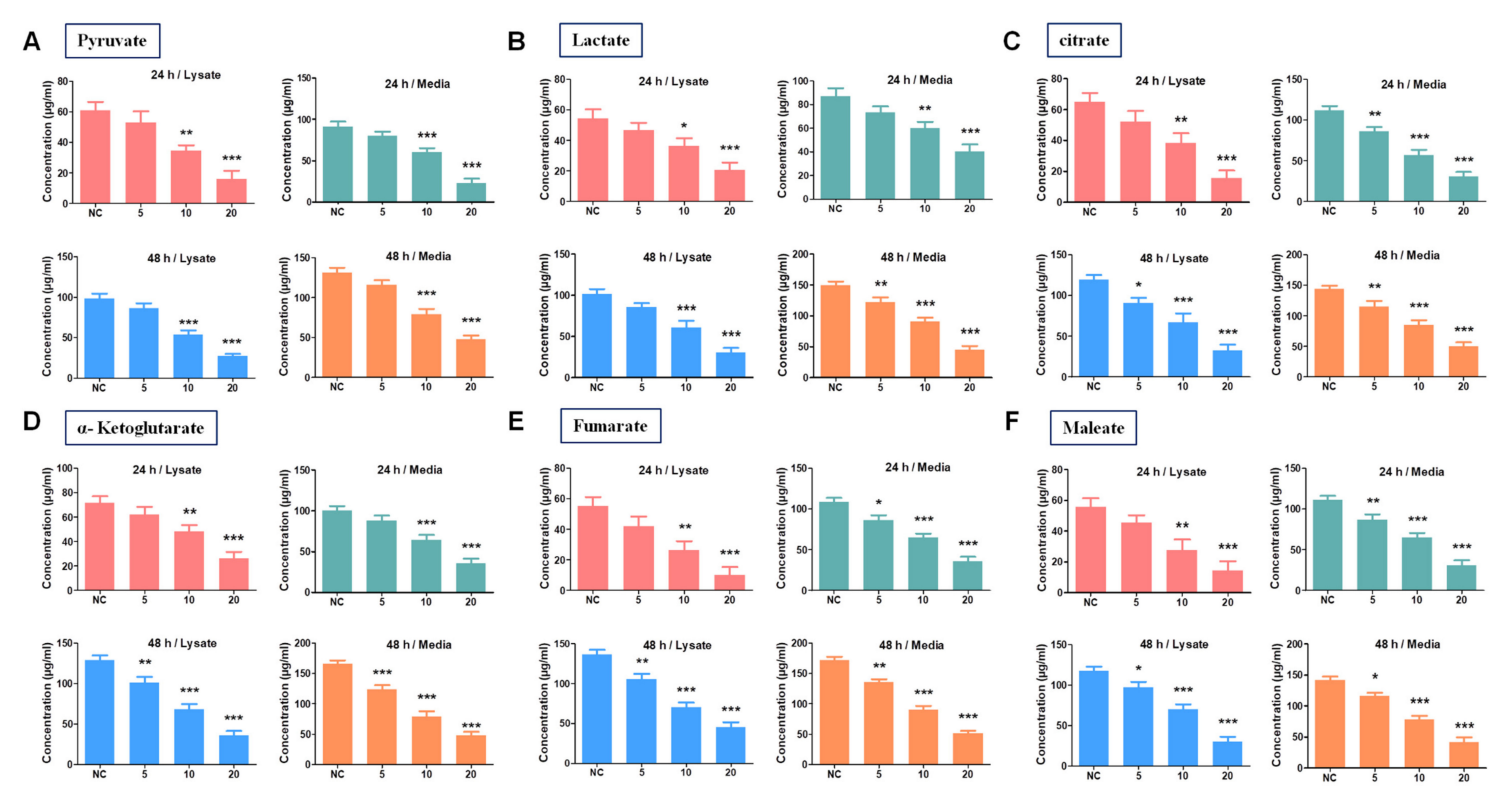
Effect of SH-859 treatment on cell metabolite level in 786-O cells after 48 h. (**A**) Quantification of pyruvate level in lysates and media. Treatment with SH-859 significantly reduced pyruvate levels in the lysate and media as compared with the normal control (untreated 786-O cells) after 48 h. (**B**) Quantification of lactate in lysates and media. SH-859 noticeably reduced the lactate level in lysates and media of 786-O cells as compared with normal control (untreated 786-O cells). (**C**) Citrate level was significantly reduced in cell lysate and media after SH-859 treatment as compared with the normal control (untreated 786-O cells). (**D**) Quantification of reduction of α-ketoglutarate concentration in the cell lysate and media was observed after SH-859 treatment, as compared with normal control (untreated 786-O cells). (**E**) Quantification of fumarate in lysates and media. SH-859 significantly reduced the concentration of fumarate in the lysate and media as compared with normal control (untreated 786-O cells). (**F**) Quantification of the level of maleate in lysates and media. SH-859 significantly reduced the concentration of maleate in the lysate and media as compared with normal control (untreated 786-O cells). Representative results from three independent experiments (*n* = 3) are shown. One-way ANOVA was used to compare the means of different groups. Differences between means were considered significant at *p* < 0.05 using Tukey’s multiple comparison test; * *p* < 0.05, ** *p* < 0.01, and *** *p* < 0.001 as compared with normal control (untreated 786-O cells).

**Figure 5:**
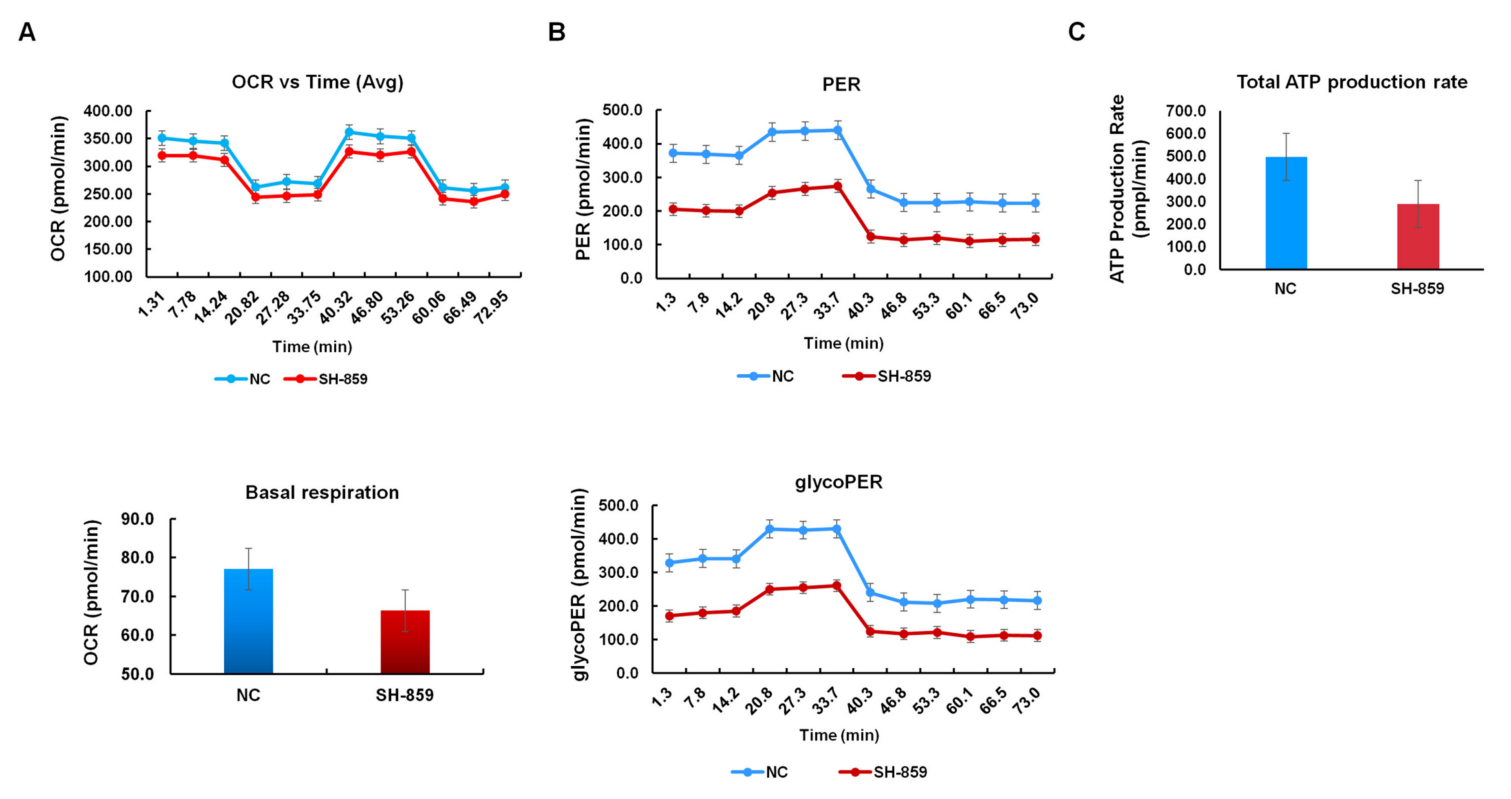
Attenuation of glycolysis in 786-O cells after SH-859 (15 μM) treatment. (**A**) No important fluctuations were noticed in the oxygen consumption rate after SH-859 treatment. SH-859 treatment inhibited basal respiration. (**B**) Proton efflux rate and proton efflux rate by glycolysis were inhibited after SH-859 treatment. (**C**) SH-859 treatment significantly declined the total ATP production rate. Representative results of three independent experiments (*n* = 3) are shown. NC: normal control (untreated 786-O cells).

To understand the alteration of aerobic glycolysis by SH-859 in 786-O cells, we examined OCR, PER, glycolytic proton efflux rate (glycoPER), and important cellular metabolite levels. SH-859 treatment reduced uptake of glucose (downregulated the expression of Glut-1; Figure 3E) and other cellular metabolites (pyruvate, lactate, citrate, α-ketoglutarate, maleate, and succinate) in the lysate and media as compared with that in the normal control (untreated 786-O cells) (Figure 4A–F) in a concentration matter. As shown by immunoblotting, the protein levels of LDHA and monocarboxylate transporter 4 (MCT4) were downregulated after SH-859 treatment (Figure 3E). The changes in cellular metabolism may be associated with a decreased level of these intercellular metabolites. SH-859 inhibited oxidative phosphorylation, as evaluated by the oxygen consumption rate (OCR) and basal respiration (Figure 5A), suggesting that SH-859 may modulate mitochondrial dynamics. Moreover, SH-859 also decreased glycolytic activity in 786-O cells, evaluated by measuring total PER and glycoPER, compared with the normal control (untreated 786-O cells) (Figure 5B). SH-859 also significantly decreased ATP production as compared with normal control (untreated 786-O cells) (Figure 5C). c-Myc, HIF-1α, and PKM2 are known to regulate aerobic glycolysis. Our results proved that SH-859 inhibited the progression of aerobic glycolysis by downregulating these proteins.

### 3.6. SH-859 Inhibited 786-O Cell Growth in a Xenograft Model

**Figure 6:**
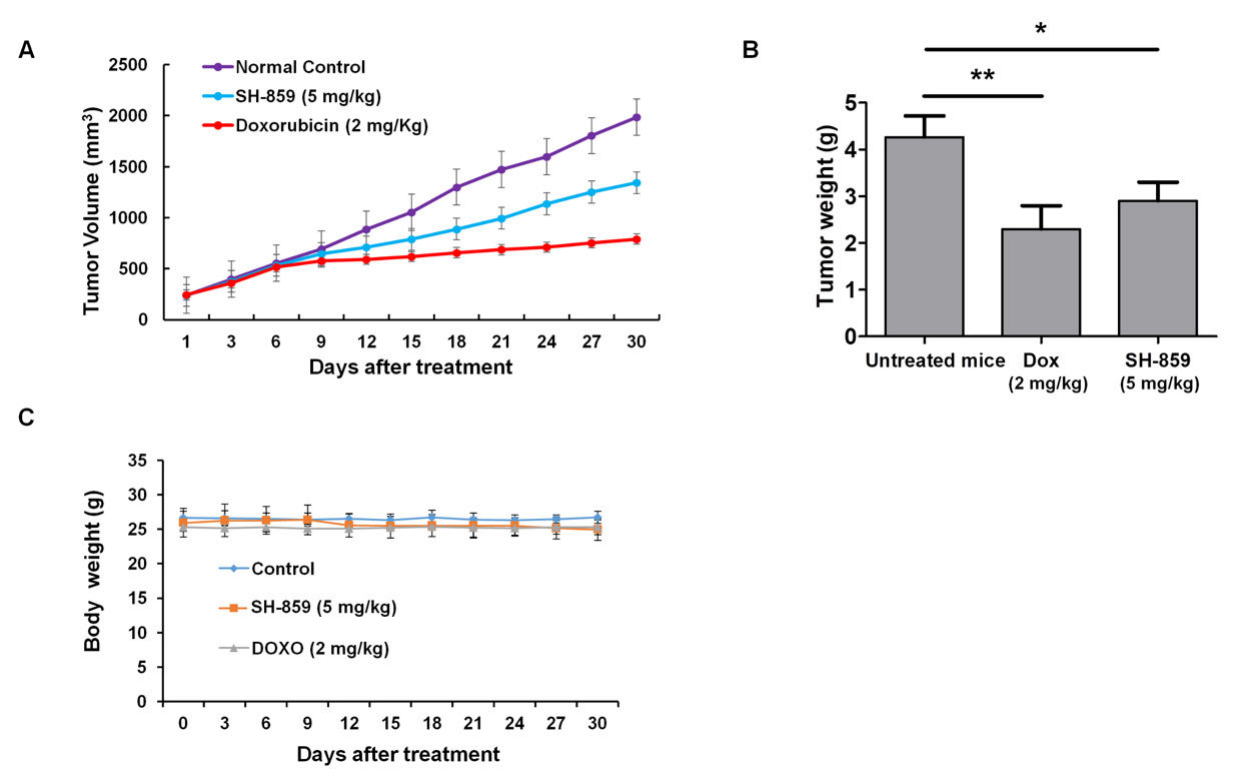
Effect of SH-859 in the tumor xenograft model. Nude mice (*n* = 5) were injected with 786-O cells intraperitoneally. Thereafter, the mice were treated with vehicle (control) (untreated mice), SH-859 (5 mg/kg, twice a week, p.o.) (test), and doxorubicin (2 mg/kg, once a week, intraperitoneally (i.p.)) (standard) for 30 days. (**A**) Tumor volume, (**B**) tumor weight and (**C**) body weight changed after treatment with SH-859 and doxorubicin. Data are represented as mean ± SE. * *p* < 0.05 and ** *p* < 0.01, compared with vehicle.

To analyze the effect of SH-859 in an in vivo xenograft model, we injected 786-O cells into nude mice. Treatment started after the tumor reached a definite size. SH-859 (5 mg/kg) p.o. or doxorubicin (2 mg/kg, i.p.) was administered for 30 days. SH-859 treatment significantly reduced tumor volume (Figure 6A) and tumor weight (Figure 6B) as compared to that in the untreated mice. No noteworthy adverse effects were detected in mice treated with SH-859. These findings suggested that SH-859 could inhibit kidney cancer cell growth in vivo.

## 4. Discussion

RCC is a common cancer of renal tubular epithelial cells and accounts for 85% of all primary renal neoplasms. A rise in the rate of incidence and mortality has been observed in RCC [37]. Novel therapeutic approaches for RCC are now in clinical trials [38,39,40]. The human immune network plays an important role in the identification and subsequent elimination of several types of cancers [41]. However, the immune network fails to attack metastatic renal cancer. Therefore, identifying a novel molecule for selective treatment against cancer is very important. Alteration of metabolism and induction of apoptosis and autophagy have been regarded as an approach to combat metastatic cancer and inhibit cancer cell proliferation and survival. Various synthetic compounds exhibit potential anticancer activity against different types of human cancers [24,26,42,43,44,45]. The present study explored the anticancer potential of SH-859 in 786-O human kidney cancer cells. SH-859 exhibited potent anticancer activities by inducing apoptosis, autophagy, and cell cycle arrest in human kidney cancer cells.

We investigated the anti-proliferative activity of the experimental small molecules (SH-763, SH-859, and SH-886) against 786-O cells by MTT assay. Here, we demonstrated that proliferation was significantly inhibited after treatment with a branched alkyl group containing oxindole derivative (SH-859), which was comparable with that in a normal kidney cell line, NRK52E. However, the other two oxindole derivatives (linear alkyl group containing derivative, SH-763, and chloro-substituted oxindole derivative containing an amide group, SH-886) also exhibited growth inhibition against 786-O cells. We also found that SH-859 not only inhibited cell proliferation but also changed cellular morphology.

The mammalian cell cycle is an extremely systematic and synchronized process that maintains the replication of genetic material and cell division [46]. It regulates different growth-regulatory signals and maintains genetic integrity to ensure no genetic damage is propagated. Abnormal cell cycle activity is a characteristic feature of cancer cells. Genetic abrasions in genes coding for cycle proteins or mutations in upstream signaling pathways can result in cancer [47]. SH-859 significantly arrested the cell cycle at the G0/G1 phase in a concentration-dependent manner. Cell cycle regulation is important for maintaining tumor growth; therefore, the anti-proliferative effect of SH-859 may result from cell cycle arrest. Blocking the cell cycle at a specific checkpoint is one of the mechanisms used for inducing cell death, and various anticancer agents do so by arresting the cell cycle at the G0/G1 or G2/M phase [48]. p21 (CDKN1A) and p27 (CDKN1B) also regulate the cell cycle protein cyclin-dependent kinase (CDK) [49]. In this study, SH-859 arrested the cell cycle at the G0/G1 phase in a dose-dependent manner by increasing the expression of p21 and p27. We found that SH-859 downregulated the expression of cyclin A, B, and D in a dose-dependent manner.

Caspase activation and modulation of cytochrome c expression (because of fluctuations in mitochondrial permeability) have been associated with the initiation of apoptosis [50]. Here, SH-859 significantly downregulated the anti-apoptotic protein Bcl-2 and upregulated the pro-apoptotic protein Bax in a concentration-dependent manner in 786-O cells. Activation of caspase 9 triggers the discharge of cytochrome c that ultimately activates the caspase pathway that blocks the upregulation or activation of proteins that help cells in repairing DNA and maintaining cellular morphology and cell cycle and initiates programmed cell death [51]. Here, SH-859 upregulated the expression of cytochrome c, which helped in the activation of apoptosis. Our results revealed that SH-859 activated apoptosis by a mitochondria-dependent pathway in 786-O cells. Flow cytometry showed that SH-859 initiated annexin V^+^PI^−^ early- and annexin V^+^PI^+^ late-stage apoptosis in 786-O cells in a concentration-dependent manner. Additionally, SH-859 upregulated cleaved caspase 3, cleaved caspase 9, and PARP, which also take part in SH-859-induced apoptosis.

In response to growth factor signaling, mTOR, downstream of phosphoinositide 3-kinase (PI3K)/AKT pathway, is typically activated. In most cases, the PI3K/AKT/mTOR pathway has been chosen as the primary target of therapy for patients with metastatic RCC [52]. Various studies have confirmed the adverse extrapolative role of low PTEN/high p-AKT/high PI3K expression in patients with RCC, which establishes the potential clinical consequence of the PI3K/AKT pathway. The role of the AKT/mTOR pathway has been well studied in cell proliferation, autophagy regulation, and tumor development [53]. Akt plays an important role in helping cells to survive under stress conditions [54], while mTOR maintains the expression of important proteins and growth factors [55]. The Akt/mTOR pathway has been seen to be activated often in RCC and is directly associated with RCC survival and progression [56,57,58,59]. Moreover, PKM2 overexpression inhibits the induction of autophagy, via activation of mTORC1 signaling, by phosphorylating AKT1S1 (an inhibitor of mTORC1) [34]. Our results indicated that SH-859 downregulates PKM2 expression, which in turn, attenuates AKT phosphorylation and subsequently downregulates phosphorylation of mTOR. We confirmed the appearance of autophagosomes and autophagolysosomes after SH-859 treatment by acridine orange and MDC staining, respectively. Furthermore, SH-859 treatment also upregulated the expression of LC3B, as seen by immunoblotting and immunocytochemistry. The expression of other autophagy-specific proteins was upregulated after SH-859 treatment.

To design anticancer therapeutics in the future, a vast knowledge of molecular crosstalk, involved in regulating cancer cell death, is essential. Both autophagy and apoptosis act together and induce cell death in a coordinated manner. For example, different autophagy proteins (e.g., Atg5 and Beclin 1) can participate in apoptotic events [60]. On the contrary, caspase-dependent cleavage of different autophagy proteins (e.g., Atg5 and Beclin 1) can also regulate autophagy [61,62]. Various proteins have been shown to play a negative regulatory role in both events [63]. Common upstream signals can trigger both apoptosis and autophagy. According to recent reports, Akt helps cell survival by inhibiting apoptosis after phosphorylating the Bcl-2 protein family member, Bad. Additionally, in cancer cells, activation of the PI3K/Akt/mTOR signaling pathway can result in inhibiting the effects of Akt on apoptosis and mTOR on autophagy, thus improving cell survival capability [64]. In this study, the simultaneous induction of both apoptosis and autophagy by SH-859 may be associated with the inhibition of the PI3K/Akt/mTOR/PKM2 signaling pathway. Further study is required to define optimal strategies involved in both apoptosis and autophagy induced by SH-859.

During tumorigenesis, an isoform change occurs that substitutes PKM1 with PKM2. This PKM2 is associated with the supply of intermediate products that are essential for the biosynthesis of nucleic acids, amino acids, and lipids in cancer cells. We found that SH-859 can inhibit the pyruvate kinase activity of PKM2 at the cell proliferation inhibitory concentration. By suggesting a role for SH-859 in inhibiting PKM2, we are not excluding the possibility that SH-859 also targets other proteins, some of which have already been recognized by Western blotting.

Both glycolysis and glycolytic proteins are highly upregulated in various cancers; cancer cells use the glycolytic pathway to synthesize their metabolites and maintain cell division and growth. We investigated the effect of SH-859 treatment on 786-O cells using the XFe96 analyzer. Cells uptake glucose to produce lactate and protons, which when transported to the extracellular medium acidify the cellular environment. SH-859 significantly reduced the rate of proton extrusion in the extracellular medium during glycolysis (glycoPER) as compared with normal control (untreated 786-O cells). Similarly, cells use glucose and other fuels in mitochondrial respiration for energy production. Thus, mitochondrial-derived CO_2_ also plays a part in extracellular acidification. Here, significant inhibition of PER was observed after SH-859 treatment, which was detected instantly after Rot/AA injection (an inhibitor of mitochondrial respiration which characteristically increases glycolysis), as compared to the normal control (untreated 786-O cells). Moreover, SH-859 treatment significantly downregulated glycolytic capacity and suppressed mito-acidification. Thus, SH-859 significantly inhibited glycolysis in 786-O cells.

To examine any possible alterations in the metabolic profile between normal control (untreated 786-O cells) and SH-859 treated cells, we measured basal mitochondrial respiration rate (OCR). SH-859 treatment caused little change in OCR, however, an increase in OCR/PER ratio was observed in the SH-859-treated cells as compared to the normal control (untreated 786-O cells) (data not shown). This suggested that a limited metabolic shift to oxidative phosphorylation occurred after SH-859 treatment. The rate of cellular ATP production is another measurement of cellular metabolism. Therefore, cellular metabolism is an extremely regulated process where cells adjust their ATP production on the basis of their ATP demand. In mammalian cells, ATP is produced by two primary metabolic pathways—glycolysis and oxidative phosphorylation. In 786-O cells, SH-859 treatment suppressed the rate of glycolysis, which resulted in the inhibition of ATP synthesis. HPLC analysis showed that SH-859 treatment significantly decreased the levels of cellular metabolites, such as pyruvate, lactate, citrate, α-ketoglutarate, maleate, and fumarate, suggesting that SH-859 altered 786-O cell metabolism.

Cellular growth can be influenced by paracrine signaling. Seeding more cells increases the growth rate as compared with a lower density population. The clonogenic assay is a 3D cell culture assay where anchorage-independent growth has been observed. Normal cells cannot grow without a substrate, whereas cancer cells can. Cancerous cells proliferate indefinitely and produce large colonies. This in vitro assay is one of the basic tools to examine the ability of cells to produce colonies (or cellular transformation) [10]. In the clonogenic assay, cells were exposed to the chemical agent for a longer time and there was no inter-cellular messaging to defend them from chemical substances. Therefore, treatment with SH-859 made these cells more vulnerable to form colonies than in the survival assay (where a high number of cells were seeded). Here, SH-859 inhibited the colony formation ability of 786-O cells as compared with the normal control (untreated 786-O cells).

Finally, SH-859 suppressed the development of tumors resulting from human kidney cancer cells in a xenograft model. The administration of SH-859 had a significant effect on tumor growth compared with the untreated mice.

## 5. Conclusions

In this study, we demonstrated the anticancer potential of SH-859, with special emphasis on its molecular mechanism against human kidney cancer 786-O cells. SH-859 induced mitochondria-mediated apoptosis, along with autophagy in 786-O cells. Inhibition of the Akt/mTOR/PKM2 signaling pathway by SH-859 could have contributed to its antitumor activity. Here, we successfully demonstrated that SH-859 not only blocked 786-O cell energy metabolism (glycolysis) but also inhibited kidney cancer cell proliferation and colony formation ability. Therefore, our data provide a rationale for developing SH-859 as an antitumor agent that targets human kidney cancer.

## Supplementary Materials

The following are available online at https://www.mdpi.com/2218-273X/10/9/1260/s1, Figure S1: Comparative cytotoxic profile (determined using MTT assay) of the small molecules (SH-763, SH-859, SH-886) against 786-O and NRK52E cells at concentrations ranging from 5–50 μM for 48 h, Figure S2: Graphical representation of the cell cycle distribution after flow cytometry analysis.
