# Supplementary figures and images for: Biological Evaluation of Oxindole Derivative as a Novel Anticancer Agent against Human Kidney Carcinoma Cells
Source: Biomolecules. 2020 Aug 31;10(9):1260. doi: 10.3390/biom10091260 (PMC7565513; doi:10.3390/biom10091260)

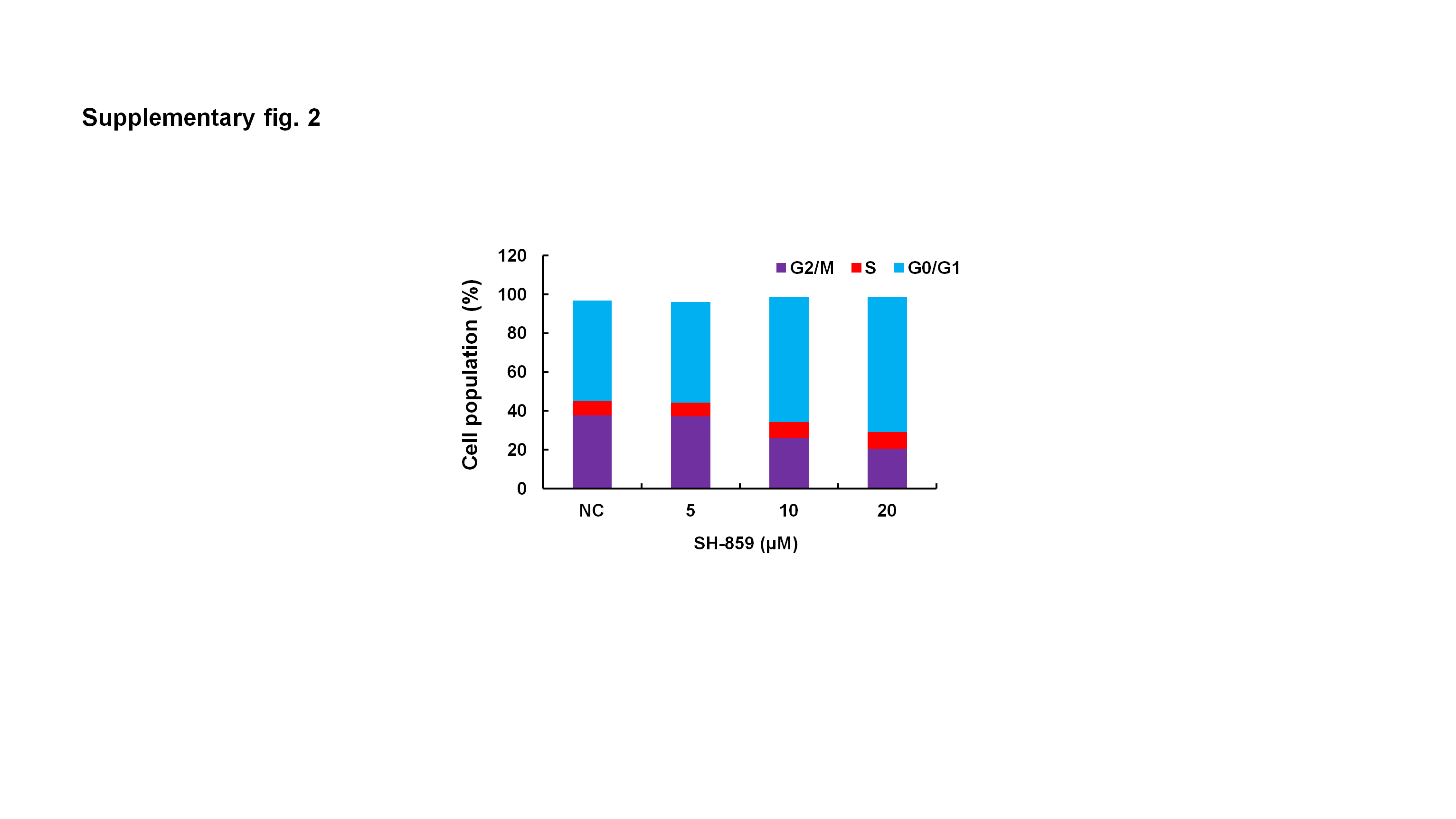

Supplement: Supplementary file 1 [file biomolecules-10-01260-s001.zip › Supplementary figure 2_Revision 2.tif]

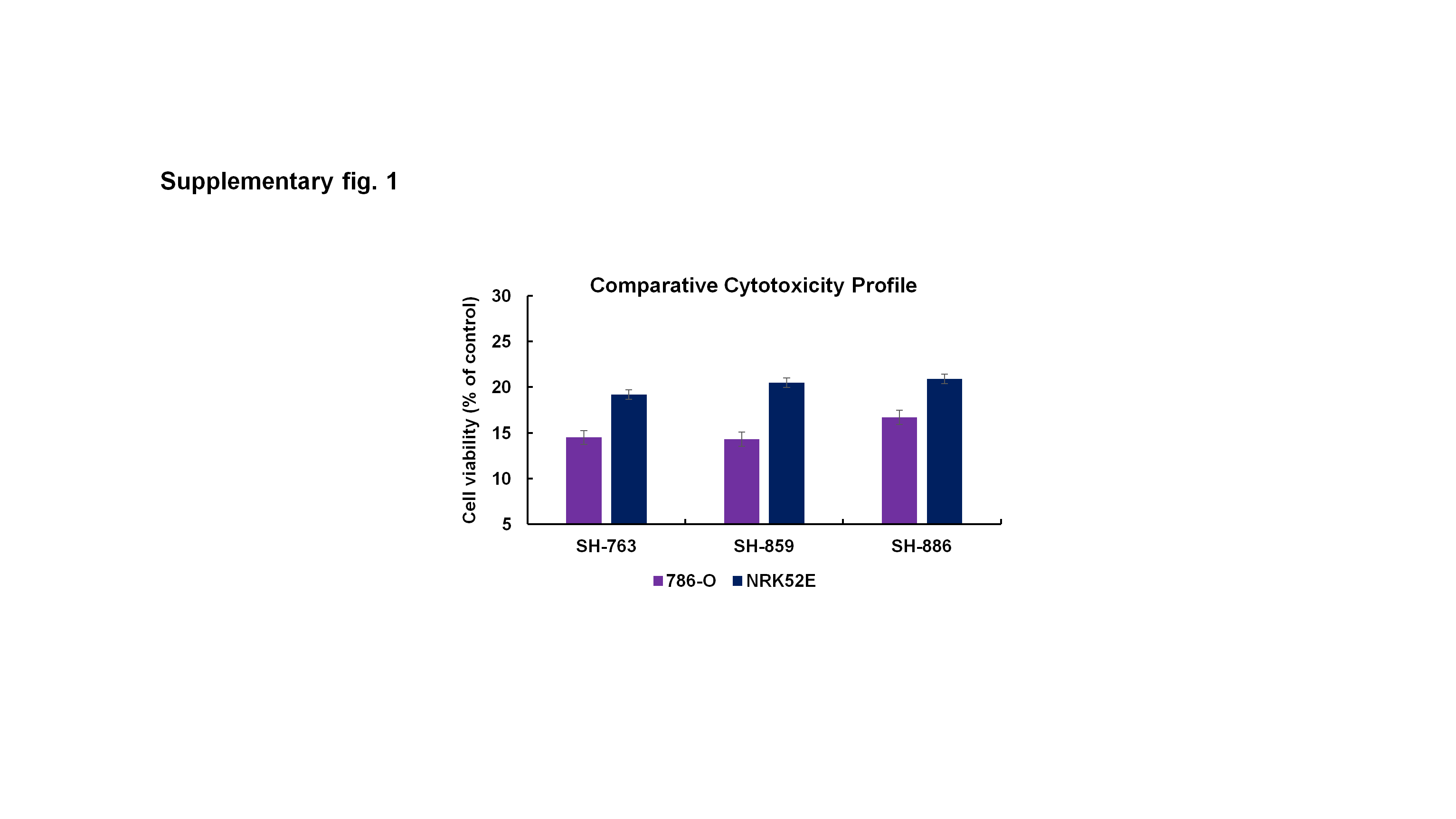

Supplement: Supplementary file 1 [file biomolecules-10-01260-s001.zip › Supplementary figure 1_Revision 2.tif]
